# Combined genotype and haplotype tests for region-based association studies

**DOI:** 10.1186/1471-2164-14-569

**Published:** 2013-08-21

**Authors:** Sergii Zakharov, Tien Yin Wong, Tin Aung, Eranga Nishanthie Vithana, Chiea Chuen Khor, Agus Salim, Anbupalam Thalamuthu

**Affiliations:** 1 Human Genetics, Genome Institute of Singapore, Singapore, Singapore; 2 Saw Swee Hock School of Public Health, National University of Singapore, Singapore, Singapore; 3 Singapore Eye Research Institute, Singapore, Singapore; 4 Department of Ophthalmology, National University Health System, Singapore, Singapore; 5 Centre for Healthy Brain Ageing (CHeBA), School of Psychiatry, University of New South Wales, Sydney, Australia

**Keywords:** Genotype-based tests, Haplotype-based tests, Association analysis, Test statistic combination

## Abstract

**Background:**

Although single-SNP analysis has proven to be useful in identifying many disease-associated loci, region-based analysis has several advantages. Empirically, it has been shown that region-based genotype and haplotype approaches may possess much higher power than single-SNP statistical tests. Both high quality haplotypes and genotypes may be available for analysis given the development of next generation sequencing technologies and haplotype assembly algorithms.

**Results:**

As generally it is unknown whether genotypes or haplotypes are more relevant for identifying an association, we propose to use both of them with the purpose of preserving high power under both genotype and haplotype disease scenarios. We suggest two approaches for a combined association test and investigate the performance of these two approaches based on a theoretical model, population genetics simulations and analysis of a real data set.

**Conclusions:**

Based on a theoretical model, population genetics simulations and analysis of a central corneal thickness (CCT) Genome Wide Association Study (GWAS) data set we have shown that combined genotype and haplotype approach has a high potential utility for applications in association studies.

## Background

The development of genotyping and sequencing technologies has enabled scientists to investigate the impact of genomic loci on complex disorders and traits. Indeed, genome-wide association studies (GWAS) and sequencing studies have identified many common single-nucleotide polymorphisms (SNPs) (for GWAS publication list, see http://www.genome.gov/gwastudies/) and rare variations [1-4] associated with common diseases. Although single-SNP analysis has proven to be useful in discovering many disease-associated loci, this strategy may be limited due to very stringent significance threshold and poor reproducibility [5]. Region-based association studies have the advantages of less stringent significance level and potentially higher power if multiple associated variants are found within a region. Indeed, several empirical studies have demonstrated the superiority of genotype gene-based association analysis over single-SNP strategy [6,7]. Also, there is some theoretical [8,9] and empirical evidence that haplotype-based tests may possess higher power than SNP-based tests. When intending to use haplotypes in an association study, one faces a problem of phase inference. While several statistical algorithms have been developed to infer unknown haplotypes from genotype data [10-12], the improvements of sequencing technologies will enable researchers to assemble haplotypes from sequencing data with very high accuracy (for examples of existing assembly algorithms, see Bansal et al. [13], Bansal et al. [14], and Schatz et al. [15]). This opens up the opportunity to use high-quality haplotypes and genotypes in sequencing association studies.

Numerous studies have reported cases when haplotype-based analysis resulted in detection of an association, while SNP-based analysis either did not yield any significant results or yielded much higher p-values [16-19]. A haplotype-based test may be more powerful than a genotype-based test if haplotypes tag a true causal variant better (although the imputation of untyped SNPs using publicly available reference panels may also be a powerful strategy), or if a SNP-SNP interaction is present within a region. In general, it is unknown whether haplotype- or genotype-based tests are more relevant for identifying an association of a genomic region with a phenotype. In this article we propose two statistical tests that explicitly combine both genotype and haplotype information for the purpose of preserving high power under both genotype and haplotype disease scenarios. We investigate two methods based on a combination of p-values from genotype- and haplotype-based association tests. The first method is a minimum of p-values (MinP-val), and the other is a sum test statistic based on inverse standard normal transformation of two p-values (SumP-val). Based on simulations, theoretical power calculations and application to a GWAS data set, we have highlighted the merits and the drawbacks of genotype- and haplotype-based tests, and those of our combined approaches. The major conclusions from our work are as follows:

1. Combination of haplotype- and genotype-based test statistics preserves power for both genotype and haplotype disease models;

2. In some of the considered scenarios, the performance of the MinP-val approach is comparable to those of the SumP-val method;

3. MinP-val is much more robust than SumP-val when one of the underlying tests has low power.

## Methods

### Genotype- and haplotype-based tests

Let us assume that we are interested in testing the joint association of all the variants within a genomic region with either a dichotomous phenotype or quantitative trait. Next, assume we have chosen the two statistical tests for a region-based association analysis: one genotype- and one haplotype-based test. For haplotype-based tests haplotypes can be inferred from genotypes [10-12] or assembled from sequencing data [13,15,20]. Several conventional genotype-based methods [21-23] are applicable for common variants testing, whereas for sequencing data numerous recently-developed rare variants approaches are available [24-28]. Haplotype-based methodologies have also been extensively published elsewhere [29-31], including rare haplotype tests [32-34].

### The combined approaches

Let us denote p-values from a genotype- and a haplotype-based tests as *p*_1_ and *p*_2_ respectively. Our first approach is SumP-val [35]. Let us consider the inverse standard normal transformation of both p-values and which are distributed as standard normal random variables under the null hypothesis. Here, we assume that *y*_1_,*y*_2_ is bivariate normal. The SumP-val test statistic is *P*_sum_ = *y*_1_ + *y*_2_. Under the null hypothesis, it is distributed as a normal random variable with zero mean and variance *Var*(*y*_1_ − *y*_2_) = *Var*(*y*_1_) + 2*Cor*(*y*_1_, *y*_2_) − *Var*(*y*_2_) = 2 + 2*p*, where *p* is a correlation coefficient between *y*_1_ and *y*_2_, since two statistical tests for the same genomic region may not be independent. The correlation coefficient *p* may be estimated via permutation procedure. The rejection region is large values of the test statistic, which is equivalent to low values for *p*_1_ and/or *p*_2_. The theoretical p-value for SumP-val test is calculated as , where *Φ*(*a*, *b*, *c*) is a value of normal cumulative distribution function with mean *b* and variance *c* taken at the point *a*.

Our second approach (MinP-val) is to utilize the minimum of the two p-values as a test statistic, namely, *min*{*p*_1_, *p*_2_} [36]. Let us represent a test statistic as *min*{*p*_1_, *p*_2_} = *min*{1 − *Φ*(*y*_1_), 1 − *Φ*(*y*_2_)}, where *y*_1_ and *y*_2_ defined above are distributed as standard normal random variables under the null. Thus, the theoretical cumulative distribution function of MinP-val test statistic under the null hypothesis can be calculated as follows:

(1) $$\begin{array}{l} p\left(\min\left\{1-\Phi \left(y_{1}\right),1-\Phi \left(y_{2}\right)\right\}<x\right)=P\left(1-\max\left\{\Phi \left(y_{1}\right),\Phi \left(y_{2}\right)\right\}<x\right)=\\ =P\left(\left(1-x\right)<\max\left\{\Phi \left(y_{1}\right),\left(y_{2}\right)\right\}\right)=1-P\left(1-x>\Phi \left(y_{1}\right),1-x>\Phi \left(y_{2}\right)\right)=\\ =1-P\left(\Phi^{-1}\left(1-x\right)>y_{1},\Phi^{-1}\left(1-x\right)>y_{2}\right), \end{array}$$

where 0 < *x* < 1. Given the rejection region is small values of the test statistic, theoretical p-value for MinP-val test is straightforward to compute using (1).

### Theoretical power model

Within our theoretical framework the following model is adopted: the two test statistics *S*_*g*_ and *S*_*h*_ of the underlying genotype- and haplotype-based tests, respectively, are assumed to asymptotically follow central chi-squared distribution $X_{1}^{2}$ with 1 degree of freedom under the null hypothesis, and non-central chi-squared $X_{1a}^{2}$ and $X_{1b}^{2}$ with NCPs *a* and *b,* respectively, under the alternative hypothesis. One of the examples of the test which results in such null and alternative distributions is Rao’s score test on, for example, genotype or haplotype scores described in Additional file 1. Since the two tests are applied to the same data, the chi-squared test statistics are likely to be positively correlated. The correlation between the two test statistics may vary from very low to high. For example, if within a region there are few SNPs in very high LD then we would expect the correlation between the tests to be high. Alternatively, we would expect the correlation to be low when variants within a region are independent. The correlation is modeled via underlying multivariate normal distribution, namely, to simulate the test statistics $S_{g}\sim X_{1a}^{2}$ and $S_{h}\sim X_{1,b}^{2}$ a bivariate normal random vector y=y1,y2 with mean $\left(\sqrt{a},\sqrt{b}\right)$, unit variances and correlation coefficient *p* > 0 is generated, and the squares of the coordinates are taken as the proxy for the test statistics: $S_{g}=y_{1}^{2}$, $S_{h}=y_{2}^{2}$. To estimate the power of MinP-val and SumP-val tests we simulated 500,000 independent pairs (*S*_*g*_, *S*_*h*_) under the alternative huypothesis, calculated the test statistics for the combined approaches, and noted the share of statistically significant pairs. This procedure was done for every theoretical scenario (see “Results” section).

### Population genetics simulations

King et al. [37] provided the SFS_CODE (http://sfscode.sourceforge.net) implementation of population genetics simulation for ANGPTL4 gene exons (http://home.uchicago.edu/~crk8e). The authors assumed the demographic and distribution fitness effect parameters from Boyko et al. [38] and Gutenkunst et al. [39]. Using the SFS_CODE program 1000 haplotype pools each containing 20000 sampled “individuals” (40000 chromosomes) from a European population were generated. A data replicate was created from each haplotype pool by iterative random sampling of two haplotypes (thus, defining the genotype of an “individual”) and assigning a dichotomous phenotype conditional on a multi-site genotype or a pair of haplotypes. Each data replicate contains 500 cases and 500 controls. Let us assume that there are L variants within the genomic region of interest, and the genotype of “an individual” $\left\{g_{1},\ldots ,g_{1}\right\}$ is constructed from the sampled haplotypes. To describe the genotype-based disease model let us, without loss of generality, denote the genotypes at rare (MAF < 1%) causal SNPs as $\left\{g_{1},\ldots ,g_{c}\right\}$, causal common SNP *g*_*c-1*_ (if present depending on a model), and other SNPs $\mathrm{as}\left\{g_{c‒2},\ldots ,g_{1}\right\}$. Let us also define the assigned odds ratios of causal variants {*b*_1_, …, *b*_*c* − 1_}. The probability of a disease *P*(*A*) for a genotype-based scenario is calculated from the following:

(2) $$\log\left(\frac{P\left(A\right)}{1-P\left(A\right)}\right)=\log\left(\frac{0.01}{1-0.01}\right)+\sum_{l=1}^{c+1}g_{l}\log\left(b_{1}\right).$$

For the haplotype-based scenarios let us consider the two sampled haplotypes {*h*_*1*_*,h*_*2*_}. Also, denote *H*_*r*_ and *H*_*c*_ as the sets of rare (frequency in a haplotype pool <1%) and common causal haplotypes, respectively (depending on the disease model *H*_*c*_ may be empty). The probability of a disease is defined as:

(3) $$\begin{array}{ll} \;\log\left(\frac{P\left(A\right)}{1-P\left(A\right)}\right)= & \log\left(\frac{0.01}{1-0.01}\right)+\sum_{i=1}^{2}\left(l\left\{h_{i}\in H_{r}\right\}\log\left(d_{r}\right)\right)\\ & \left(+l\left\{h_{i}\in H_{c}\right\}\log\left(d_{c}\right)\right), \end{array}$$

where *l*{*A*} is an indicator of an event *A*, and {*d*_*r*_*,d*_*c*_} are the odds ratios for causal rare and common haplotypes, respectively. For our simulations, we considered three phenotype models: “Rare” (only rare variants or haplotypes are risk-contributing), “Common” (only common variants or haplotypes), and “Both” (both types of variants or haplotypes). Following the scenarios of exome-scale simulations of Wu et al. [40], we have assigned 50%, 20% and 10% of the observed rare variants (haplotypes) to be causal. Additionally, we chose one common causal SNP (haplotype) for “Both” and “Common” models. The odds ratios ${\left\{b_{l}\right\}}_{l=1}^{c-1}$ in (2) and {*d*_*r*_*,d*_*c*_} in (3) were assigned as follows:

– for the “Rare” model: *b*_*l*_ = *d*_*r*_ = 4*,l* = 1*,..,c* and *b*_*c*-1_ = *d*_*c*_ = 0

– for the “Both” model: *b*_*l*_ = *d*_*r*_ = 3*,l* = 1*,..,c* and *b*_*c*-1_ = *d*_*c*_ = 1.2

– for the “Common” model: *b*_*l*_ = *d*_*r*_ = 1.5*,l* = 1*,..,c* and *b*_*c*-1_ = *d*_*c*_ = 2

The average number of variants across data replicates is shown in Table 1.

**Table 1:** The average number of variants within a region across 1000 data replicates in population genetics simulations

**Phenotype model**	**Proportion of causal variants/haplotypes**		
	**50%**	**20%**	**10%**
Haplotype common	32.4	31.6	31.6
Haplotype both	35.5	33.2	32.6
Haplotype rare	37.2	34.1	33.0
Genotype common	33.1	32.4	32.2
Genotype both	36.3	33.7	32.9
Genotype rare	37.6	34.4	33.0

### Real data analysis

For the purpose of demonstrating the performance of the described methodologies we conducted a gene-based analysis of the central corneal thickness (CCT) GWAS data sets described in Vithana et al. [41]. Briefly, the Singapore Indian Eye Study (SINDI), which is part of the Singapore Indian Chinese Cohort Eye Study (SICC) [42], consists of 2538 Indian subjects aged 40 and above, and the Singapore Malay Eye Study (SiMES) [43-45] is a genome-wide association study of CCT phenotype which contains 2542 Malay subjects aged 40 and above. Both SiMES and SICC adhered to the Declaration of Helsinki. Ethics approval for the both studies was obtained from the Singapore Eye Research Institute Institutional Review Board [41]. The combined data set consists of 5080 individuals genotyped at 552318 SNPs after quality control. In total, 5049 individuals were analyzed after excluding those with missing phenotype. Also, we attempted to replicate all the genome-wide significant regions using Chinese samples from the SICC. This data set contains 2837 samples with non-missing phenotype and covariates (age and gender). SNPs were mapped to genes based on the method outlined by Zhao et al. [46]. Briefly, information on gene identifiers (IDs), names, start and end positions on a chromosome were downloaded from the NCBI Genome database (http://www.ncbi.nlm.nih.gov/Genomes). Gene regions included 10 kb upstream and downstream. Hierarchical mapping scheme (coding > intronic > 5′UTR > 3′UTR) was used if a variant was within 10 kb of multiple genes. The remaining inter-gene variants between two genes were grouped together. Haplotype inference was performed using the software Beagle [10] with reference panel from 1000Genomes Project (http://www.1000genomes.org/). In our analysis we adjusted for age, gender and the first ten principal components from Eigenstrat [47].

### Statistical tests for population genetics simulations and real data application

Sequence Kernel Association Test (SKAT), introduced by Wu et al. [40], is a variance component score test derived from a semi-parametric regression model. It was initially proposed to test the association of phenotype with multi-site genotype; however, we also used SKAT to test an association of phenotype with haplotypes as described below. To show the consistency of empirical results we applied the same pair of underlying tests, namely, genotype SKAT and haplotype SKAT with linear kernel and uniform weights, to both population genetics simulations and real data. For genotype SKAT all rare variants (MAF < 1% in the sample) within a region were collapsed according to the method described by Thalamuthu et al. [48]. Briefly, the collapsed super-variant is the sum of minor alleles across rare variants within a region; if this sum is greater than 2 then the value of 2 is assigned. We did not apply weighting as described by Wu et al. [40] because that would substantially decrease power to identify an association with common SNPs. Haplotype-based SKAT is SKAT applied to a haplotype regression matrix *R,* which is constructed similar to those used by Zaykin et al. [31]. First, we pooled all rare haplotypes into one haplotype group, whereas each of the common haplotypes formed a separate haplotype group. Let us define the following notations: *n* is a number of individuals; {*H*_1_,…,*H*_*M*_} is the haplotype groups with *H*_*M*_ being the most common group; $R={\left\{R_{\mathrm{ij}}\right\}}_{\mathrm{ij}=1}^{n,M-1}$ is a haplotype regression matrix; and {*h*_*i,*1_;*h*_*i,*2_} is a pair of haplotypes for *i* th individual. The haplotype matrix {R_*ij*_} is constructed as follows:

(4) $$\begin{array}{ll} R_{i\;j} & =l\left\{h_{i,1}\in H_{j}\right\}+l\left\{h_{i,2}\in H_{j}\right\},i=1,\ldots ,n;j\\ & =1,\ldots ,M-1, \end{array}$$

where *l*{*A*} is an indicator of an event *A*. If there were no common haplotypes within a region, we formed three groups of haplotype: those with a frequency less that 0.05%, those in between 0.05% and 0.1%, and those with a frequency greater than 0.1%. For both tests we used the R (http://www.r-project.org/) package SKAT (http://www.hsph.harvard.edu/~xlin/software.html). For population genetics simulations p-values for all the tests were estimated using 1000 permutations. In real data analysis for the underlying tests we used theoretical p-values as we believe that they reasonably approximate empirical p-values given large sample size and normally-distributed quantitative trait [41]. Then we tested an assumption of bivariate normality by applying the Shapiro-Wilk test (R package “mvnormtest” http://cran.r-project.org/web/packages/mvnormtest/). If the normality test was not significant on the genome-wide level, we used theoretical p-values for both SumP-val and MinP-val; otherwise we used permutations. The permutation procedure and estimation of correlation coefficient are described in the next section.

### Permutation procedure and estimation of correlation coefficient

To calculate theoretical p-values for the proposed methods we estimated the correlation coefficient *p* using 500 permutations. The difficulty in applying permutations lies in the fact that the permutation procedure should preserve the relationship between all the covariates, and also between phenotype and covariates, but disrupt the relationship between phenotype and genotype. Several techniques have been developed for conducting permutation tests of partial coefficients in a multiple regression model [49-51]. Among them the permutation of residuals under the reduced model [49] was shown to preserve correct type-1 error for t-test [52] and was previously applied to microarray data analysis [53]. As the SKAT test can be obtained from a semi-parametric regression model [40], let us consider the following genotype and haplotype regression models: *Y* = *a*_1_ − *f*_1_(*P*) − *Cc* − *ϵ* and *Y* = *a*_2_ − *f*_2_(*R*) − *Cc* − *ϵ*, where *P* is *n* × *L* collapsed genotype matrix, *n* is the sample size, *L* is the number of common SNPs within a region plus one for collapsed rare variants super-locus, *Y* is *n* × 1 vector of quantitative phenotype (CCT), *C* is *n* × 12 matrix of covariates which include age, gender and the first ten genotype principal components obtained from Eigenstrat [47], *R* is haplotype regression matrix, and *f*_1_ and *f*_2_ are unknown functions. To obtain the permutation values for the test statistics the reduced model *Y* = *a* − *Cc* − *ϵ* is fitted, and *a*, *c*, *ϵ* are the estimated constant coefficient, regression coefficients and residuals, respectively. Next, the residuals *ϵ* are permuted to obtain *ϵ*, and *Y* = *a* + *Cc* − *ϵ*. The permuted statistic values for both genotype and haplotype SKAT tests are calculated as respective SKAT statistics from semi-parametric models *Y* = *a*_1_ − *f*_1_(*P*) − *Cc* − *ϵ* and *Y* = *a*_2_ − *f*_2_(*R*) − *Cc* − *ϵ*. Each p-value obtained from permutations was transformed using the inverse standard normal transformation, and the value of *p* was estimated by a Pearson correlation coefficient.

## Results

### Theoretical power results

**Figure 1:**
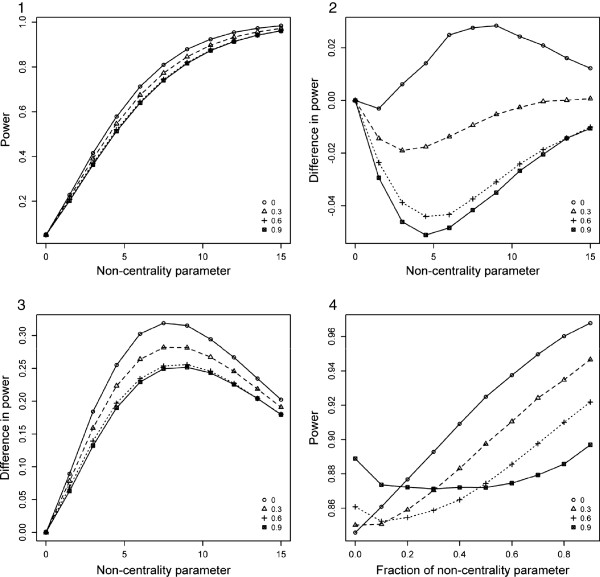
**Performance of MinP-val approach under the theoretical models.** Different lines across the panels correspond to different levels of correlation *p* - 0, 0.3, 0.6 and 0.9. **Panel 1**: Power of MinP-val test as a function of NCP *a* and correlation *p*; **Panel 2**: Difference in power between MinP-val and the more powerful underlying test as a function of NCP *a* and correlation *p*; **Panel 3**: Difference in power between MinP-val and the less powerful underlying test as a function of NCP *a* and correlation *p*; **Panel 4**: Power of MinP-val test as a function of correlation *p* and the ratio of NCP *b* to NCP of the more powerful underlying test (the latter is fixed at 10.5).

**Figure 2:**
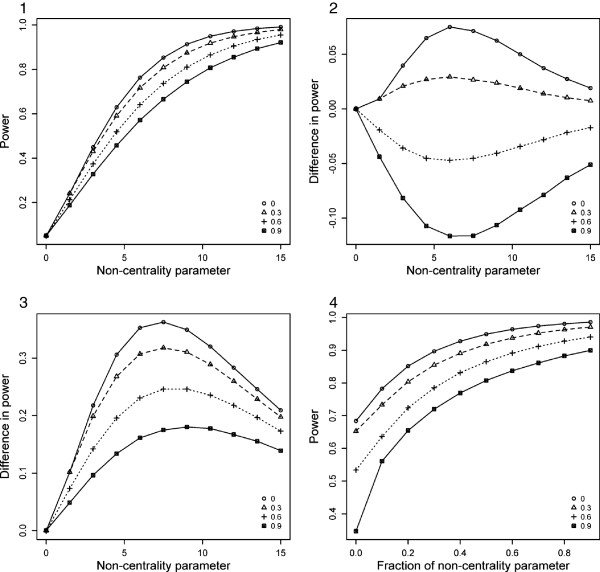
**The performance of SumP-val approach under the theoretical models.** Different lines across the panels correspond to different levels of correlation *p* - 0, 0.3, 0.6 and 0.9. **Panel 1**: Power of SumP-val test as a function of NCP *a* and correlation *p*; **Panel 2**: Difference in power between SumP-val and the more powerful underlying test as a function of NCP *a* and correlation *p*; **Panel 3**: Difference in power between SumP-val and the less powerful underlying test as a function of NCP *a* and correlation *p*; **Panel 4**: Power of SumP-val test as a function of correlation *p* and the ratio of NCP *b* to NCP of the more powerful underlying test (the latter is fixed at 10.5).

Depending on the disease model, one of the underlying tests (genotype- or haplotype-based) is expected to be more powerful than the other underlying test. So, we assume that under the alternative hypothesis the non-centrality parameter (NCP) of the more powerful underlying test is , and the NCP of the less powerful underlying test is *b* = a/2. Figures 1 and 2 (Panel 1) show the power of MinP-val and SumP-val strategies as a function of correlation coefficient *p* and NCP *a* at the fixed type-1 error of 0.05. As can be seen in Panel 1, power in general decreases slightly with increasing correlation. Panel 2 depicts the difference in power between the combined approaches and the more powerful underlying test. It is notable that both MinP-val and SumP-val achieved greater power than the more powerful underlying test for lower correlation. Also, MinP-val approach lost a maximum of 5% power for high correlation and gained a maximum of 2% for low correlation, whereas for SumP-val these values are more than 5% in both cases.

In Panel 3, where the difference in power between the combined approaches and the less powerful underlying test is shown, it can be seen that both MinP-val and SumP-val are consistently better than the less powerful test. This suggests that combination of statistical tests may prove beneficial when the underlying disease model is unknown. To investigate the impact of change of NCP *b* on the performance of the proposed approaches we fixed NCP *a* to be equal to 10.5 (corresponding to 90% power of a chi-squared test with $X_{1a}^{2}$ distribution under the alternative hypothesis, the type-1 error is 0.05). Panel 4 of Figures 1 and 2 depicts the power of MinP-val and SumP-val as a function of correlation and a “fraction of NCP” – the ratio of *b* to 10.5. As can be seen in Panel 4, MinP-val test achieved higher power than SumP-val in the majority of scenarios. It is notable that SumP-val lost much power when the value of *b* is low. Hence, MinP-val approach is more robust with respect to underperformance of one of the underlying tests.

### Population genetics simulation results

**Figure 3:**
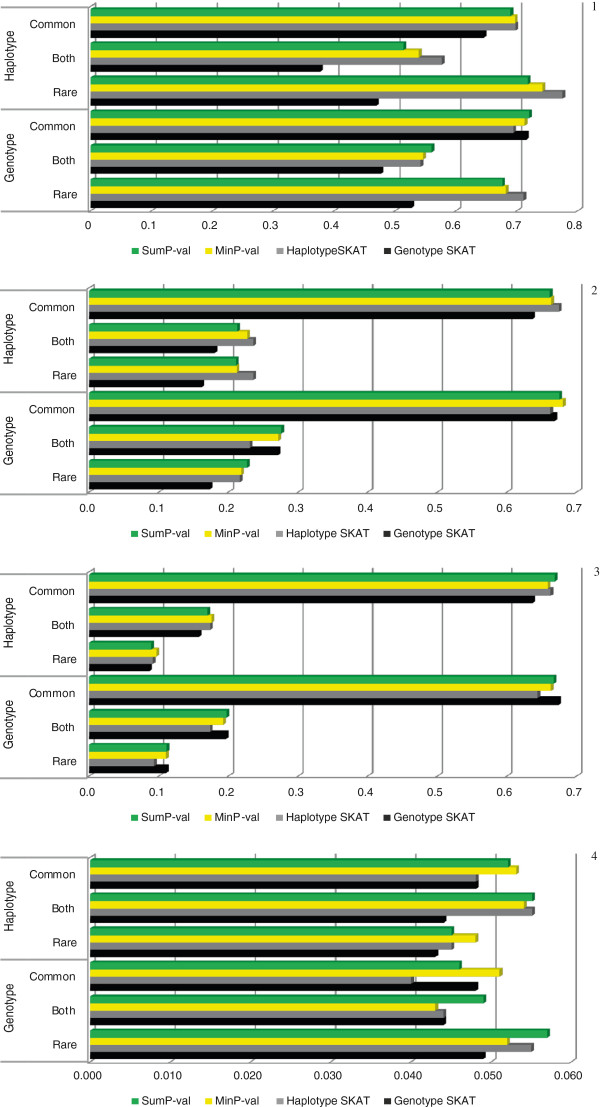
**Power comparison of genotype-based SKAT, haplotype-based SKAT, MinP-val and SumP-val tests for population genetics simulations, and an estimate of empirical type-1 error.** In each panel the top three disease models correspond to the haplotype-based disease scenario, whereas the lower three correspond to the genotype-based scenario. Disease models “Rare”, “Both” and “Common” are described in the section “Population genetics simulation”. Type-1 error is set to 5%. **Panel 1**: 50% of rare variants/haplotypes were assumed to be causal; **Panel 2**: 20% of rare variants/haplotypes were assumed to be causal; **Panel 3**: 10% of rare variants/haplotypes were assumed to be causal; **Panel 4**: empirical type-1 error estimate for simulations under the null hypothesis.

Panel 4 of Figure 3 shows the empirical type-1 error estimate for the theoretical level of 0.05 for all the tests. The estimate of type-1 error is distributed as a binomial random variable with 1000 trials and the probability of success 0.05 under the hypothesis of no inflation. The one-sided 99% quantile of the described distribution is 0.067. As can be seen, in our simulations the type-1 error was well controlled for all the tests.

Panles 1–3 of Figure 3 depict the results of population genetics simulations analysis for all the phenotype models with 50%, 20% and 10% or rare causal variants/haplotypes, respectively, at the fixed 5% type-1 error. For all the tests 1000 permutations were performed to estimate p-values. Haplotypes were assumed to be known without ambiguity. Under the genotype-based disease scenarios, genotype SKAT is expected to be more powerful than haplotype SKAT, and vice versa under the haplotype-based scenarios. However, genotype SKAT was less powerful for many genotype-based phenotype models. A possible explanation of this observation is that when rare variants are strongly associated with phenotype, for some statistical tests pooling of rare haplotypes may be a better strategy than pooling of rare variants. Also, it should be noted that with the decrease in the percentage of causal rare variants/haplotypes, the power for “Rare” and “Both” phenotype models decrease substantially since for these models rare variants/haplotypes are the major carriers of an association signal. For “Common” phenotype model one common variant/haplotype has a significant impact on phenotype; so, the decrease in power with the lower proportion of causal rare variants/haplotypes is not as high as for other phenotype models.

As can be seen from the Panels 1–3 of Figure 3, for all the phenotype disease models, when both underlying tests were almost equally powerful (e.g. Panel 1 haplotype disease scenario “Common” model, and genotype disease scenario “Both” and “Common” models), the power of both MinP-val and SumP-val were on the same level or even higher than those of the underlying tests. However, when genotype-based SKAT significantly underperformed haplotype-based SKAT (e.g. Panel 1 haplotype disease scenario “Rare” and “Both”models), MinP-val approach showed slightly lower power than the more powerful underlying test and greater power compared with SumP-val approach. The maximum power loss of SumP-val and MInP-val compared with the more powerful underlying test across all phenotype models was 6.3% and 3.8% respectively (haplotype disease scenario “Both” model). These results are consistent with those obtained from the theoretical power considerations, and illustrate the great potential of the proposed methods in their application to real association studies.

To examine the effect of phasing on our results we repeated the analysis using the most probable haplotypes inferred by Beagle [10]. The reference panel consisted of 1094 simulated individuals to mimic the size of the publicly available reference panel from the 1000 Genomes Project (http://www.1000genomes.org). The results of this analysis were very similar to those described above (data not shown). In addition, we applied the proposed methods with a different pair of underlying tests. The results are similar to those described above. For more details, see Additional files 1 and 2.

### Application to central corneal thickness GWAS data set

A total of 552318 SNPs were mapped using the hierarchical mapping algorithm described in the “Methods” section. As a result, we obtained 36146 genes and between-gene blocks. Regions that reached genome-wide significance (1.38E-6 after Bonferroni correction) for at least one of the four applied tests are presented in Table 2. We identified all of the significant regions reported by Vithana et al. [41]: COL8A2 gene, an interval between the genes RXRA and COL5A1 and a region near ZNF469 gene. As can be seen from Table 2, genotype-based SKAT and MinP-val tests achieved genome-wide significance for all the five regions listed, whereas SumP-val failed to reach genome-wide significance for both RXRA-COL5A1 and C7orf42 regions. This highlights that MinP-val approach performed better than the SumP-val approach. Haplotype-based SKAT failed to identify any association signal for four out of the five regions. From our results it is clear that for this data set genotypes were more relevant for identifying associated regions. Judging from the performance of the Haplotype SKAT there is no evidence of association of haplotypes with a phenotype except for COL8A2 gene. It is also of interest to compare our results to those reported by Vithana et al. [41] for single-SNP analysis. For the two out of three regions genotype-based SKAT and MinP-val methods outperformed the single-SNP analysis and yielded lower p-values, which may be explained by the utilization of linkage disequilibrium (LD) within a region to boost power. Given the sensitivity of SumP-val approach to underperformance of one of the underlying tests, this method showed higher p-values. To justify our assumption of bivariate normality for calculation of p-values for the proposed tests, we used the Shapiro-Wilk test. The corresponding p-values for the significant regions are presented in the (Additional file 1: Table S1) in the first row. All the p-values, except those for COL8A2-TRAPPC3 region, are non-significant at the genome-wide level, which suggests there is no evidence against the bivariate normality assumption. The Shapiro-Wilk test of COL8A2-TRAPPC3 region yielded a marginally significant p-value on the genome-wide level. Hence, the p-value for this region in the Table 2 is based on permutations.

**Table 2:** The results of the combined SiMES and SINDI data analysis and the single-SNP p-values from the original article

	**COL8A2**	**ZNF469-LOC100128913**	**RXRA-COL5A1**	**COL8A2- TRAPPC3**	**C7orf42**
Chromosome	1	16	9	1	7
Number of SNPs	4	27	73	3	6
Genotype SKAT	**3.68E-13**	**2.13E-15**	**4.06E-12**	**2.63E-08**	**2.55E-07**
Haplotype SKAT	**5.58E-10**	0.149394	0.79	2.78E-05	0.005
MinP-val	**3.68E-13**	**4.22E-15**	**8.11E-12**	**4E-08**	**4.96E-07**
SumP-val	**1.77E-11**	**9.44E-10**	7.90E-06	**4E-08**	2.60E-05
Single-SNP analysis*	rs96067: 5.4E-13	rs9938149: 1.63E-16 rs12447690: 1.92E-14	rs1536478: 3.5E-9	-	-

Genome-wide significant p-values for gene-based tests are shown in bold. * SiMES and SINDI meta analysis p-values from Vithana et al. [41].

Table 3 shows the results of the replication analysis. For the region COL8A2-TRAPPC3 the reported replication p-value is based on permutations, whereas for other regions there was no evidence against bivariate normality assumption (Additional file 1: Table S1, second row). As can be seen from Table 3, only RXRA-COL5A1 region was significant after Bonferroni correction for all the tests except for the haplotype-based SKAT. Our replication results are consistent with those of Cornes et al. [54] who found strong evidence of association of multiple SNPs within the RXRA-COL5A1 region, and marginal significance of COL8A2 SNP rs96067. It is worth noting that in our analysis C7orf42 gene, which was not identified by Vithana et al. [41], reached genome-wide significance in SiMES + SINDI data set and had moderate p-value in the replication dataset. Cornes et al. [54] found this gene to be significant in a meta-analysis of SiMES, SINDI, 1883 samples from the Singapore Chinese Eye Study and 798 samples from the Beijing Eye Study [55]. The role of C7orf42 gene in central corneal thickness (CCT) phenotype requires further investigation. The results of our analysis suggest that RXRA-COL5A1 region may have an impact on CCT phenotype.

**Table 3:** Replication results on Chinese samples from the Singapore Indian Chinese cohort eye study

	**COL8A2**	**ZNF469-LOC100128913**	**RXRA-COL5A1**	**COL8A2- TRAPPC3**	**C7orf42**
Genotype SKAT	0.019	0.117	**0.001**	1	0.014
Haplotype SKAT	0.599	0.479	0.1	1	0.27
MinP-val	0.037	0.223	**0.002**	0.989	0.028
SumP-val	0.089	0.186	**0.001**	0.788	0.027
Single-SNP analysis*	rs96067: 0.036	rs9938149: 0.4 rs12447690: 0.03	rs1536478: 0.016	-	-

Significant p-values are shown in bold. For single-SNP analysis the Bonferroni correction corresponds to the four tests. * Trend test within a linear regression model.

In addition to the gene-based analysis, we tried to replicate the four genome-wide significant SNPs found by Vithana et al. [41] in our Chinese samples using single-SNP analysis. Having tested an association of these SNPs with CCT trait using trend test within a linear additive model adjusting for age, gender and the first ten principal components, we found that none of the SNPs was significant on the corrected type-1 error rate 0.0125 = 0.05/4. This result suggests that gene-based replication may be a more powerful strategy than single-SNP replication.

In addition to the main genome-wide analysis of SiMES + SINDI data set, we applied the proposed methods with a different pair of underlying tests to the three regions reported by Vithana et al. [41]. Both MinP-val and SumP-val identified the three regions on genome-wide significance level. This result suggests that our combined approaches work as well with other underlying tests (for more details, see Additional file 1).

## Discussion

When the underlying disease model is unknown, combining statistical tests tailored for different disease scenarios may be a much better strategy than application of a statistical test designed for one specific disease model. In this article we have described the two approaches of combining genotype- and haplotype-based statistical tests. The results of theoretical power considerations, population genetics simulations and real data analysis showed strong performance of MinP-val approach for different disease scenarios, whereas SumP-val method was shown to perform poorly when one of the underlying tests had low power. Our analysis of SiMES + SINDI identified the three regions found by Vithana et al. [41], and additionally, the C7orf42 gene. The replication analysis confirmed an association of RXRA-COL5A1 region, which is consistent with the results of Cornes et al. [54], and showed a moderate p-value for C7orf42 gene. The analysis of real data highlighted the applicability of our combined approaches to real association studies.

In our simulations the Haplotype SKAT was the most powerful test in many cases, but in real data analysis it performed the worst. It is not known beforehand whether a genotype- or a haplotype-based test would perform better; hence, our proposal to apply a combined approach is a robust choice. Indeed, MinP-val did well in both simulations and real data. This emphasizes the major point of the combined strategy: MinP-val may have slightly lower power when a disease model fits Haplotype SKAT and higher power when the disease model is closer to the second underlying tests. One of the possible reasons for the apparent inconsistency of Haplotype SKAT performance may be that for “Rare” and “Both” simulation models we assumed that rare variants bear the major association signal whereas in the real data only common SNPs were present. However, Haplotype SKAT performed well even for “Common” model when a common SNP was causal. We suppose that for this scenario genotype association translated into an association of haplotypes with a phenotype, which is possible if common SNPs within a region are in high LD with each other. On the other hand, if a causal common SNP within a region is in low LD with other common SNPs within a region then under a genotype-based disease scenario haplotype-based test may have much lower power than a genotype-based test which is observed in the results of the real data analysis.

The methods proposed in this study may be easily generalized to multiple statistical tests, namely, instead of two underlying tests it is possible to apply more tests and combine all of them via the described methodology. In this case the arguments for theoretical p-value calculation for the proposed approaches can be extended in a straightforward manner.

Recently Derkach et al. [56] investigated the performance of the combined approaches, namely, the minimum of p-values and the Fisher p-value combination, for rare variants association scenarios. Although the approaches we propose are similar, our major idea is different. We combine two test statistics for the purpose of widening the set of alternatives for which our test is powerful; thus, we choose the underlying tests designed for very different phenotype models, whereas Derkach et al. [56] used linear and quadratic tests which are likely to be both powerful under many models. As a result, our conclusions are different from those of Derkach et al. [56]. For example, the authors stated that “hybrid test statistics provide much needed robustness in terms of power for association tests”, whereas we observed that only minimum p-value approach really preserves power when one of the underlying tests underperforms. Secondly, the authors found that in many cases Fisher method outperforms both of the underlying tests, and the minimum p-value approach. However, from our work it is clear that SumP-val (which is similar to the Fisher p-value combination) outperforms all the three tests only when both of the underlying tests have comparable power which is unlikely if the two underlying tests are deliberately chosen to fit very different phenotype models.

One of the limitations of the proposed approaches is the need to use permutations. For theoretical p-value calculation both SumP-val and MinP-val require a correlation coefficient to be estimated via permutations. Moreover, permutations need to be applied when asymptotic distributions of the underlying test statistics are unknown or inadequate to describe the empirical distributions.

The described methodologies may be extended to preserve power under other disease models. For example, the combination of rare-variants and common-variants statistical tests applied to a sequenced region may preserve high power when either only rare or only common variants are associated with a phenotype. However, it is not known how the combined approaches will perform if both common and rare variants are associated with phenotype.

## Conclusions

In this study we have investigated the performance of combined haplotype- and genotype-based tests for the purpose of preserving high power under both genotype and haplotype disease scenarios. Based on theoretical power calculations, population genetics simulations and analysis of the real data set we have illustrated high performance and potential utility of combined approaches for association studies.

## Competing interests

The authors declare that they have no competing interests.

## Authors’ contributions

SZ, AS and AT conceived the study. SZ and AT designed the experiments. SZ conducted the experiments and performed the analysis. TYW, TA, ENV, and CCK provided the GWAS data. SZ and AT wrote the manuscript. SZ, TYW, TA, EV, KCC, AS and AT approved the manuscript.

### Funding

This work was supported by the Agency for Science, Technology and Research (A*STAR), Singapore. The first author is a recipient of the Singapore International Graduate Award.

## Supplementary Material

Additional file 1: Table S1 Shapiro-Wilk bivariate normality test p-values for the genome-wide significant genes, additional simulation and real data analysis results. Shapiro-Wilk test was used in real data analysis to justify our assumption of bivariate normality for calculation of theoretical p-values for MinP-val and SumP-val tests. Additional simulations and real data analysis were performed using different pair of underlying tests. Click here for file

Additional file 2 **Power comparison of the gene score haplotype test, the gene score genotype test, MinP-val and SumP-val statistical tests for population genetics simulations, and an estimate of empirical type-1 error.** In each panel the top three disease models correspond to the haplotype-based disease scenario, whereas the lower three correspond to the genotype-based scenario. Disease models “Rare”, “Both” and “Common” are described in the section “Population genetics simulation”. Type-1 error is set to 5%. Panel 1: 50% of rare variants/haplotypes were assumed to be causal; Panel 2: 20% of rare variants/haplotypes were assumed to be causal; Panel 3: 10% of rare variants/haplotypes were assumed to be causal; Panel 4: empirical type-1 error estimate for simulations under the null hypothesis. Click here for file
