# Supplementary material for: Combined genotype and haplotype tests for region-based association studies
Source: BMC Genomics. 2013 Aug 21;14:569. doi: 10.1186/1471-2164-14-569 (PMC3852120; doi:10.1186/1471-2164-14-569)
Supplement: Additional file 1: Table S1 — Shapiro-Wilk bivariate normality test p-values for the genome-wide significant genes, additional simulation and real data analysis results. Shapiro-Wilk test was used in real data analysis to justify our assumption of bivariate normality for calculation of theoretical p-values for MinP-val and SumP-val tests. Additional simulations and real data analysis were performed using different pair of underlying tests. [file 1471-2164-14-569-S1.docx]

**Table S1 – P-values for the Shapiro-Wilk bivariate normality test for genome-wide significant genes in real data analysis.**

| Shapiro-Wilk test | COL8A2 | ZNF469-LOC100128913 | RXRA-COL5A1 | COL8A2- TRAPPC3 | C7orf42 |
| --- | --- | --- | --- | --- | --- |
| SiMES+SINDI | 0.08 | 0.07 | 0.73 | 1.36E-6 | 0.04 |
| Replication dataset | 0.229 | 0.149 | 0.228 | 3E-3 | 0.43 |

**Results of additional simulation analysis**

We investigated the performance of the proposed strategies for a different pair of underlying tests. For a genotype-based test we used a Gene Score test described in Zhao and Thalamuthu [[1](#_ENREF_1)] with Madsen-Browning weights [[2](#_ENREF_2)] calculated across all the samples. Briefly, for a $n\times L$ genotype matrix $G$ (SNPs in columns), vector of weights $w=(w_{1},\ldots,w_{L})$ and $n\times1$ vector $Y$ of dichotomous phenotype, the logistic model $logit\left( Y \right)=a+\left( Gw \right)b$ is considered. The genotype Gene Score test is a t-test of $b$ coefficient for the null hypothesis $H_{0}:b=0$. The haplotype Gene Score test is a t-test of the respective coefficient in a logistic regression of phenotype against the haplotype score. The haplotype score of an “individual” is the sum of Madsen-Browning weights (calculated from haplotype frequencies across all the samples) corresponding to the two haplotypes.

Panel 4 of Additional file 2 shows the empirical type-1 error estimate for the theoretical level of 0.05 for all the tests. As can be seen, in our simulations the type-1 error was well controlled for all the tests. Panels 1-3 of Additional File 2 depict the results of population genetics simulations analysis for all the phenotype models with 50%, 20% and 10% or rare causal variants/haplotypes, respectively, at the fixed 5% type-1 error. The haplotypes were assumed to be known without ambiguity. We also performed the same analysis with haplotypes inferred with Beagle using the reference panel of 1094 individuals to mimic the size of the publicly available reference panel from the 1000 Genomes Project (www.1000genomes.org) —and the results were very similar (data not shown). As can be seen from Additional File 2 for genotype scenarios, the genotype Gene Score test performed better or equally good compared with the haplotype Gene Score test, whereas for haplotype risk scenarios, the result was the opposite, except for the “Common” phenotype model. This may be explained by the fact that the frequency of some common haplotypes may be very high (for example, wild type haplotype); so, if a very common haplotype is chosen to confer risk, it will be underweighted too much in the haplotype Gene Score test. Since both Gene Score tests were designed to account for the potential effect of rare variants or rare haplotype, the relative power of the tests under common disease scenarios may not follow the expectations. It is also notable that the MinP-val test was on par with the SumP-val method for all the phenotype models, except when one of the underlying tests significantly underperformed the other underlying test. In these cases, MinP-val performed better than SumP-val, which is consistent with the conclusions obtained for the different pair of the underlying tests.

**Results of additional real data analysis**

In addition to the main genome-wide analysis of the SiMES+SINDI data set, we applied different pair of underlying tests and our proposed methods to the three regions reported by Vithana et al. [[3](#_ENREF_3)]. For the genotype-based test we utilized the regression on principal component (PC) scores of genotype [[4](#_ENREF_4)]. To describe the methodology, let us denote $G$ as $n\times L$ genotype matrix, where $n$ is the sample size and $L$ is the number of SNPs within a region, $Y$ is $n\times1$ vector of quantitative phenotype, $C$ is $n\times12$ matrix of covariates which include age, gender and the first ten genotype principal components obtained from Eigenstrat [[5](#_ENREF_5)]. Further, let us define the $n\times p$ matrix $P$ as a matrix with columns being principal component scores obtained from $G$. The matrix $P$ contains the minimum number of principal components with the cumulative variance no less than 80% of the total variance [[4](#_ENREF_4)]. In other words, the principal components in order from highest to lowest variance were recursively added to the matrix $P$ until the sum of variances of the columns exceeded 80% of the total variance (sum of variances of all principal components). This procedure reduces the number of variables while preserving the major share of genotype variability. Further, the following regression model was considered:

|  | $Y=a+Pb_{1}+Cc+\varepsilon$ | (1) |
| --- | --- | --- |

where $a$ is the constant term, $b$ and $c$ are $p\times1$ and $12\times1$ vectors of regression coefficients, and $\varepsilon$ is $n\times1$ vector of error terms. A statistic to test the null hypothesis $H_{0}:b_{1}=0$ is the F-statistic:

|  | $F_{1}=\frac{(SSR-SSF)/p}{SSF/(n-p-13)}$ | (2) |
| --- | --- | --- |

where $SSF$is the sum of squared residuals in the full model (3), and $SSR$ is the sum of squared residuals in the reduced model $Y=a+Cc+\varepsilon$. Under the null hypothesis the test statistic $F_{1}$ is asymptotically distributed as F random variable with $p$ and $n-p-13$ degrees of freedom as the CCT phenotype is a normally distributed trait [[3](#_ENREF_3)].

For the haplotype-based test we applied the regression on haplotype clusters obtained from the affinity propagation algorithm [[6](#_ENREF_6)]. Clustering of haplotypes is needed to reduce the degrees of freedom of F-statistic and to overcome the difficulty of analyzing rare haplotypes within a regression framework. Affinity propagation is a clustering algorithm built on the idea of exchanging real-valued messages between data points until “a high quality set of exemplars and corresponding clusters gradually emerge” [[7](#_ENREF_7)]. The input of the algorithm requires a similarity matrix $\left\{ s\left( i,j \right) \right\}_{i,j=1}^{N}$, where, for $i\neq j$the element $s\left( i,j \right)$ is a measure of how well the data point $j$ is suited to be an exemplar for the data point $i$, and for $i=j$ the element $s(i,i)$ is a measure of likelihood of the data point $i$ to be an exemplar (cluster center). Let us assume we have $h$ unique haplotypes $\{H_{k}, k=1,\ldots,h\}$ for a region (a haplotype $H_{k}$ can be written as vector $\left\{ x_{k1},x_{k2},\ldots,x_{kL} \right\}, x_{kl}\in\{0,1\}$). The order of markers on a haplotype is assumed to be the physical order on the chromosome. To construct $h\times h$ haplotype similarity matrix $s(i,j)$ Jin et al. [[6](#_ENREF_6)] utilized the following measure:

|  | $s\left( i,j \right)=-\sum_{l=1}^{L} \frac{1}{p(x_{jl})}\left\vert log\left( \frac{p\left( x_{il} \right)}{p\left( x_{jl} \right)} \right) \right\vert, i\neq j$ | (3) |
| --- | --- | --- |

where $p\left( x_{il} \right)=P\left( x_{il}|x_{il-1} \right)$ is the likelihood of the observed allele on the haplotype $H_{i}$ on the place $l$ conditional upon the observation of an allele on the place $l-1$ (this model corresponds to the first-order Markov chain model suggested by Jin et al. [[6](#_ENREF_6)]). These probabilities are estimated using the inferred haplotypes across all the individuals. The elements $s(i,i)$ are equal to the median of values $s\left( i,j \right), i\neq j,$ which corresponds to the default setting of the ‘apcluster’ function in the affinity propagation R (www.**r**-project.org/) package “apcluster” (http://www.psi.toronto.edu/index.php?q=affinity%20propagation). For COL8A2 gene we forced the algorithm to output two clusters as the initial run gave only one haplotype cluster. Next, let us assume that all the $h$ haplotypes are split into $k$ clusters $S_{1},\ldots, S_{k}$, where we let the cluster $S_{k}$ to be the most frequent (assigned to be the reference cluster). The $n\times(k-1)$ regression matrix $R=\left\{ R_{ij} \right\}_{i=1,j=1}^{n,k-1}$ is constructed as follows: value of $R_{ij}$ is the number of haplotypes of $i$th individual that belong to cluster $S_{j}$. After the construction of $R$ matrix the following regression model is considered:

|  | $Y=a+Rb_{2}+Cc+\varepsilon$ | (4) |
| --- | --- | --- |

where $b_{2}$ is $(k-1)\times1$ vector of regression coefficients. The test statistic $F_{2}$ for the null hypothesis $H_{0}:b_{2}=0$ is analogous to (4) where $SSF$ is computed for the regression model (6). The asymptotic distribution of $F_{2}$ is F-distribution with $k-1$ and $n-\left( k-1 \right)-13$ degrees of freedom.

The permutations of residuals under the reduced model were applied to estimate the correlation $\rho$ between the inverse standard normal transforms of theoretical p-values of the underlying tests. To justify our assumption of bivariate normality we applied the Shapiro-Wilk test. The corresponding p-values for the three regions are presented in the Table S2. All the p-values are non-significant at 5% type-1 error which suggests there is no evidence against the assumption of bivariate normal distribution.

Table S3 shows the theoretical p-values for the described genotype and haplotype tests, for MinP-val and SumP-val approaches. As can be seen, in spite of haplotype-based test yielding high p-values, both of the proposed methods performed on par with the genotype-based test. It is notable that the single-SNP p-values reported by Vithana et al. [[3](#_ENREF_3)] are more significant than all the other tests considered. However, gene-based analysis reduces the number of tests from 552318 (genome-wide significance level 9.053E-8) to 36146 —number of genes and between-gene blocks in a data set (genome-wide significance level 1.38E-6), which implies the results obtained here are also significant on the genome-wide level.

**Table S2 – Additional real data analysis: p-values for the Shapiro-Wilk test.**

|  | COL8A2 | ZNF469-LOC100128913 | RXRA-COL5A1 |
| --- | --- | --- | --- |
| ShapiroWilk bivariate normality test on $(N_{1}^{*},N_{2}^{*})$ | 0.4938 | 0.7668 | 0.6285 |

**Table S3 – Additional real data analysis: the results of the real data analysis and the single-SNP p-values (SiMES and SINDI meta-analysis) from the original article.**

|  | COL8A2 | ZNF469-LOC100128913 | RXRA-COL5A1 |
| --- | --- | --- | --- |
| Genotype test p-value | 2.43E-12 | 3.65E-12 | 1.22E-08 |
| Haplotype test p-value | 0.9013 | 9.27E-06 | 4.05E-05 |
| MinP-val | 4.85E-12 | 7.30E-12 | 2.45E-08 |
| SumP-val | 1.61E-11 | 4.52E-12 | 2.34E-09 |
| Single-SNP analysis from Vithana et al. [[3](#_ENREF_3)] | rs96067: 5.4E-13 | rs9938149: 1.63E-16  rs12447690: 1.92E-14 | rs1536478: 3.5E-9 |

**References**

1. Zhao J, Thalamuthu A: **Gene-based multiple trait analysis for exome sequencing data**. *BMC Proceedings* 2011, **5**(Suppl 9):S75.

2. Madsen BE, Browning SR: **A groupwise association test for rare mutations using a weighted sum statistic**. *PLoS Genet* 2009, **5**(2):e1000384.

3. Vithana EN, Aung T, Khor CC, Cornes BK, Tay W-T, Sim X, Lavanya R, Wu R, Zheng Y, Hibberd ML *et al*: **Collagen-related genes influence the glaucoma risk factor, central corneal thickness**. *Human Molecular Genetics* 2011, **20**(4):649-658.

4. Gauderman WJ, Murcray C, Gilliland F, Conti DV: **Testing association between disease and multiple SNPs in a candidate gene**. *Genetic Epidemiology* 2007, **31**(5):383-395.

5. Price AL, Patterson NJ, Plenge RM, Weinblatt ME, Shadick NA, Reich D: **Principal components analysis corrects for stratification in genome-wide association studies**. *Nat Genet* 2006, **38**(8):904-909.

6. Jin L, Zhu W, Guo J: **Genome-wide association studies using haplotype clustering with a new haplotype similarity**. *Genetic Epidemiology* 2010, **34**(6):633-641.

7. Frey BJ, Dueck D: **Clustering by passing messages between data points**. *Science* 2007, **315**(5814):972-976.
