# Supplementary figures and images for: Combined genotype and haplotype tests for region-based association studies
Source: BMC Genomics. 2013 Aug 21;14:569. doi: 10.1186/1471-2164-14-569 (PMC3852120; doi:10.1186/1471-2164-14-569)

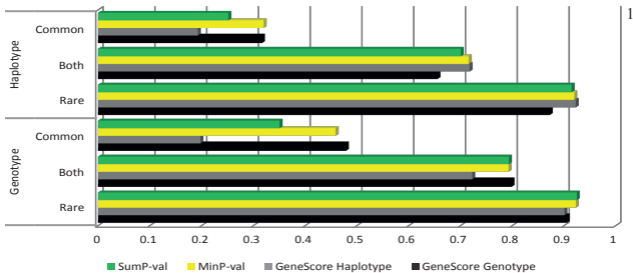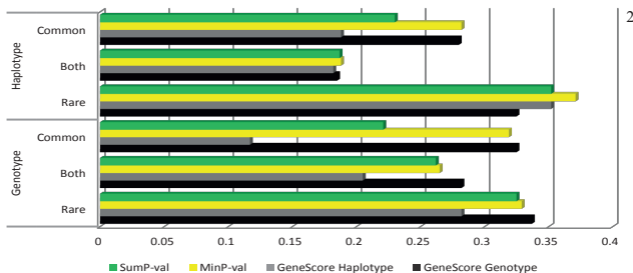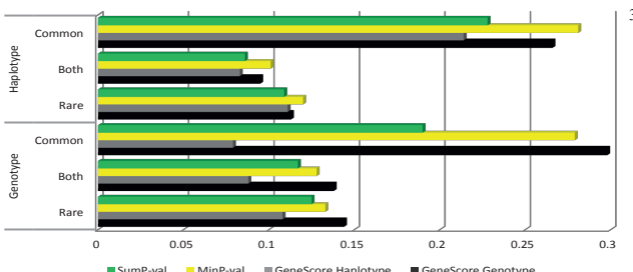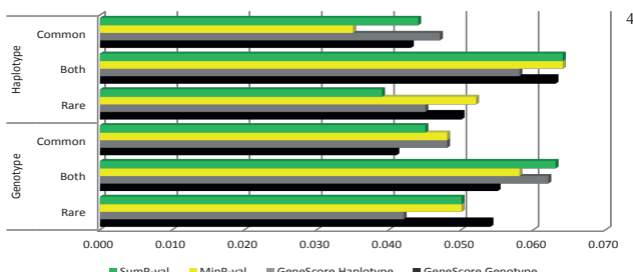

Supplement: Additional file 2 — Power comparison of the gene score haplotype test, the gene score genotype test, MinP-val and SumP-val statistical tests for population genetics simulations, and an estimate of empirical type-1 error. In each panel the top three disease models correspond to the haplotype-based disease scenario, whereas the lower three correspond to the genotype-based scenario. Disease models “Rare”, “Both” and “Common” are described in the section “Population genetics simulation”. Type-1 error is set to 5%. Panel 1: 50% of rare variants/haplotypes were assumed to be causal; Panel 2: 20% of rare variants/haplotypes were assumed to be causal; Panel 3: 10% of rare variants/haplotypes were assumed to be causal; Panel 4: empirical type-1 error estimate for simulations under the null hypothesis. [file 1471-2164-14-569-S2.pdf]
